# Bioaugmentation as microbiome engineering: a framework for evaluating functional performance, persistence, and safety

**DOI:** 10.3389/fmicb.2026.1835468

**Published:** 2026-05-11

**Authors:** Mariusz Cycoń

**Affiliations:** Department of Microbiology, Faculty of Pharmaceutical Sciences, Medical University of Silesia, Sosnowiec, Poland

**Keywords:** bioaugmentation, biodegradation pathways, biomonitoring, functional persistence, microbial consortia, microbiome engineering

## Abstract

Bioaugmentation is increasingly applied to enhance the degradation of contaminants in environmental and engineered systems, yet its effectiveness is often evaluated using endpoints that do not establish whether the introduced microbial function was expressed, sustained, or responsible for the observed outcome. This review considers bioaugmentation as an approach to microbiome engineering rather than a discrete inoculation step. It outlines an evaluation framework based on three complementary criteria: functional performance, functional persistence, and safety. Emphasis is placed on the microbiological determinants of successful implementation, including pathway completion, expression of terminal transformation steps, functional redundancy, ecological compatibility, and the maintenance of introduced activity under variable environmental conditions. The analysis also examines how inoculum design, delivery, selective pressure, retention, and biomonitoring influence the establishment of degradative functions within complex microbial communities. A central theme is the need to distinguish genuine inoculum-driven effects from outcomes generated by biostimulation, modified mass transfer, or changes in contaminant bioavailability. Available evidence indicates that robust interpretation requires an integrated assessment of substrate-to-product relationships, functional and activity markers, community-level responses, and, where appropriate, indicators of biological effect. Microbiological safety considerations are also reviewed, including selective pressures and resistance-related consequences in systems exposed to biologically active contaminants. Bioaugmentation should therefore be evaluated as a controlled microbiotechnological intervention in which function, persistence, mechanistic attribution, and safety are assessed collectively.

## 1 Introduction

Bioaugmentation refers to the deliberate introduction of selected microorganisms into environmental or engineered systems to enhance biodegradation and maintain biological process stability (Bala et al., 2022; Ebsa et al., 2024; Bhaskaralingam et al., 2025; Kumar Dixit et al., 2025; Krishna Raj et al., 2026). In contrast to biostimulation, which modifies environmental conditions, bioaugmentation introduces a specific metabolic capability when degradation is limited by insufficient abundance or activity of key microorganisms (Herrero and Stuckey, 2015; Liu et al., 2024; Devarajan et al., 2025; Gupta et al., 2026). The literature indicates that bioaugmentation should not be understood simply as the addition of microorganisms; instead, it functions as a strategy for engineering microbiome behaviour in complex and dynamic systems (Lyon and Vogel, 2011; Sites, 2021; Chettri et al., 2024). This understanding shifts attention away from viewing bioaugmentation as a single inoculation event towards the deliberate design and optimisation of microbial ecosystem function over time.

The relevance of bioaugmentation has grown, particularly for persistent and toxic contaminants for which a decrease in the concentration of the parent compound does not necessarily indicate detoxification. Many studies have reported that effective remediation requires complete transformation to a final, nontoxic product, rather than partial degradation that results in the accumulation of harmful intermediates (Major et al., 2002; Sahith et al., 2026). Hence, bioaugmentation is applied in soil and water remediation, wastewater and sludge treatment, and in the removal of substances that are resistant to degradation in standard biological systems (Hassanshahian et al., 2014; Herrero and Stuckey, 2015; Nzila et al., 2016; Tondera et al., 2021; Chen L. et al., 2025; Huang et al., 2025). Its application is expanding to complex mixtures and structurally diverse contaminant groups, including pesticides, pharmaceuticals, and waste-derived compounds, where overall microbial functional capacity is more critical than the breakdown of a single compound (Maldani et al., 2022; Werheni Ammeri et al., 2023a; Angeles-De Paz et al., 2025; Cycoń et al., 2025; Doolotkeldieva et al., 2025; Klim et al., 2026).

Evidence shows that bioaugmentation performs effectively only when the selected inoculum is compatible with the prevailing environmental and operational conditions, including salinity, aeration, substrate porosity, and organic carbon availability (Kansour and Al-Mailem, 2023; Helmy and Kardena, 2024; Baghaie and Keshavarzi, 2025; Du et al., 2025). In many systems, complete execution of degradation pathways requires the concurrent addition of co-substrates and interventions that increase contaminant bioavailability (Deng et al., 2022; Li et al., 2023). For pesticides, performance depends not only on the choice of microorganisms but also on their ability to remain active under fluctuating soil conditions; this frequently necessitates combined strategies, such as integrating bioaugmentation with sorptive materials or complementary biological treatments (Yang H. et al., 2021; Mali et al., 2022; Anwar et al., 2024; Faridy et al., 2024; Sethi et al., 2025). For pharmaceutical micropollutants, evaluation must address both the biotransformation mechanism and treatment-induced shifts in microbiome structure and function. In systems exposed to antibiotics, or to pharmaceuticals with antibiotic-like activity, this includes selection for antimicrobial resistance, changes in mobile genetic elements, and disturbance of core treatment functions (Cycoń et al., 2019; Yang Q. et al., 2021; Ferreira et al., 2023; Moghaddam et al., 2023; Sesay et al., 2025). For hydrophobic and semi-volatile contaminants, effectiveness is increasingly evaluated using exposure-based metrics, such as reductions in emissions, rather than relying only on concentration changes in the matrix (Bako et al., 2022). Enhanced degradation can also modify the toxicity profile of contaminant mixtures, and measured removal efficiencies do not always correspond to reductions in toxicity (Adrion et al., 2016). Moreover, although increasing bioavailability can affect mineralisation, combining such measures with inoculation does not always produce additive outcomes (Villaverde et al., 2019).

Evaluating bioaugmentation requires more than examining changes in contaminant concentrations; it also demands elucidation of the mechanisms underlying functional activity (Cycoń, 2026). Bradley and Chapelle (2010) reported the need to monitor functional biomarkers after inoculation, and other authors have indicated that examining specific genes and microbial populations associated with defined transformations is necessary to verify that the introduced inoculum is responsible for the observed effect (Smidt and de Vos, 2004; Scheutz et al., 2008). Advanced omics and biomonitoring methods are now used to differentiate between the mere detection of degradative capacity and its confirmed activity and persistence (Ciric et al., 2010; Mansouri et al., 2022; May et al., 2022; Varsha et al., 2022; Cycoń, 2026). Simultaneously, predictive approaches based on artificial intelligence are being developed to estimate performance and risk; however, they require robust datasets and rigorous monitoring standards to prevent excessive simplifications (Ashkanani et al., 2024; Siddique et al., 2025; Zhang et al., 2025). In practice, this need is increasingly addressed with multi-criteria decision frameworks that combine removal efficiency with toxicity, exposure, operational feasibility, and cost, for example life-cycle assessment linked to multi-criteria decision analysis for process optimisation and technology selection (Zhao et al., 2020; Zhao et al., 2026).

This review examines bioaugmentation as a microbiotechnological intervention designed to introduce or reinforce defined microbial functions in environmental and engineered systems. It proposes a framework for evaluating bioaugmentation through three complementary criteria: functional performance, functional persistence, and safety. Particular emphasis is placed on the microbiological foundations of successful outcomes, including pathway completion, evidence of activity rather than gene presence alone, integration of introduced microorganisms into resident communities, and the ecological and operational factors that determine whether function is maintained over time. The review also considers how inoculum design, delivery, retention, selective pressure, and biomonitoring support reliable attribution of observed effects to inoculation rather than to biostimulation or altered contaminant availability. Because the aim was to integrate heterogeneous evidence into an engineering-relevant assessment framework rather than to estimate a single pooled effect size, a meta-analysis was not pursued. The review does not claim exhaustive coverage of all published work; instead, it emphasises mechanistic plausibility, monitoring rigour, and the alignment of reported endpoints with risk-relevant outcomes. Evidence is synthesised thematically into a structured set of evaluation criteria and monitoring safeguards. The manuscript proceeds from evaluation logic to design logic. Section 2 defines the three criteria used to judge bioaugmentation outcome, namely functional performance, functional persistence, and safety. Section 3 summarises the mechanistic basis of pathway execution and ecological integration. Section 4 then translates that mechanistic basis into engineering decisions on inoculum selection, delivery, retention, and sustaining function. Section 5 examines how these decisions are constrained by matrix properties, Section 6 addresses monitoring and causal attribution, and Section 7 focuses on environmental safety and secondary risk.

## 2 A framework for evaluating bioaugmentation success

**Figure 1:**
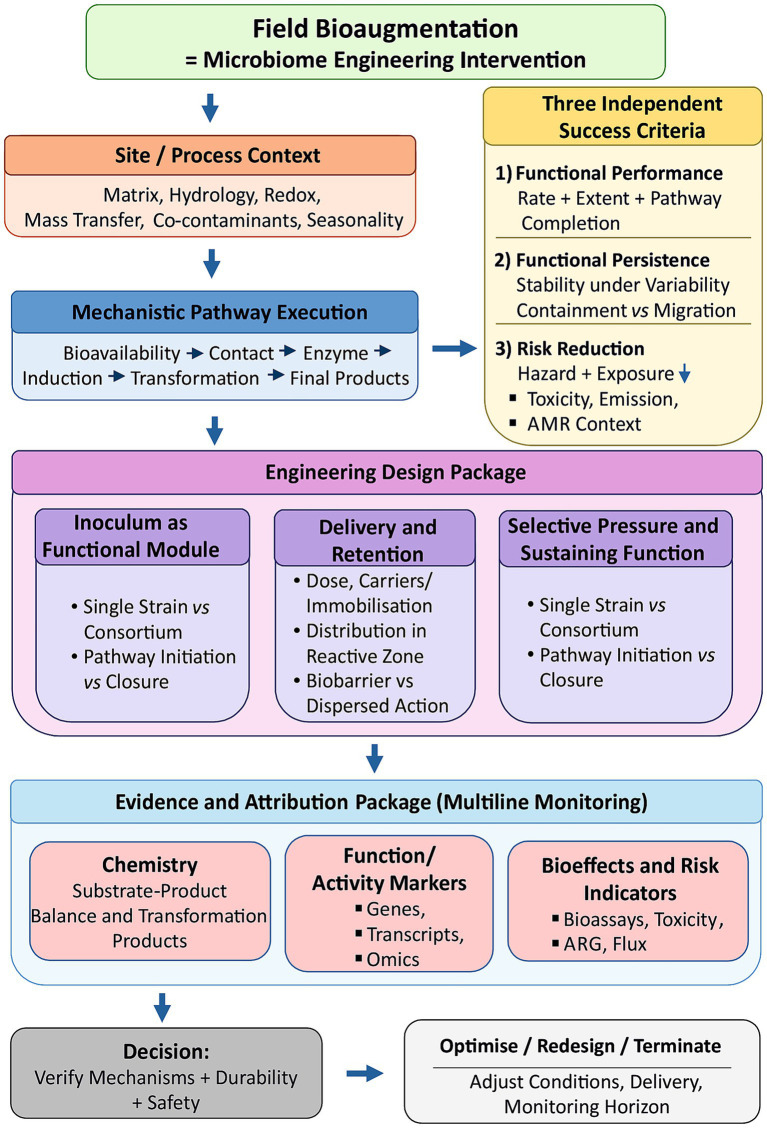
Risk-centred field bioaugmentation, from site context to mechanism, design, evidence, and decision. The figure presents field bioaugmentation as a microbiome engineering intervention organised in a defined sequence. Site and process context first determine whether the target pathway can operate under matrix, redox, hydrological, mass-transfer, and co-stressor constraints. Mechanistic pathway execution then defines the biological requirements that guide inoculum selection, delivery, retention, and maintenance of selective pressure. Evaluation is based on three criteria, functional performance, functional persistence, and risk reduction. Attribution relies on coordinated monitoring of substrate-product balances, transformation products, functional and activity markers, bioeffect measures, and exposure metrics, with controls that separate inoculation effects from operational effects.

In practice, bioaugmentation is rarely implemented in isolation. It is always combined with operational measures such as adding electron donors, modifying redox conditions, providing co-substrates, increasing hydrophobic compound bioavailability, or regulating aeration (Lyon and Vogel, 2011; Chettri et al., 2024; Sagar and Priti, 2025; Gupta et al., 2026;). The resulting outcome therefore reflects both the activity of the introduced inoculum and the environmental conditions that affect its persistence, pathway expression, and competitiveness within the ecosystem. Distinguishing bioaugmentation from biostimulation is therefore crucial, even though this distinction is often overlooked. Without separating these processes, results may be misinterpreted and the transferability of observed effects to field conditions cannot be assessed reliably (Pimmata et al., 2013; Herrero and Stuckey, 2015; Huang et al., 2025; Krishna Raj et al., 2026). Clear differentiation allows the identification of whether the outcome is attributable to the introduced inoculum, modified process conditions, or their interaction. Therefore, bioaugmentation should be evaluated on the basis of three independent criteria, namely effectiveness of transformation, persistence of the effect, and reductions in environmental and health risks. Applying these criteria enables the attribution of outcomes to their underlying mechanisms and supports reliable assessment of performance under practical conditions. Figure 1 provides an overview of the proposed framework by linking site context, mechanistic pathway execution, engineering design, evidence generation, and decision criteria.

### 2.1 Process performance: degradation rate, extent, and pathway completion

Operationally, the effectiveness of bioaugmentation is defined by the rate and extent of removal of the target compound or group of compounds and, where exposure depends on emissions, by changes in emission flux. In chloroethene-contaminated systems, a decrease in concentration is insufficient if intermediate products accumulate. In their field studies, Major et al. (2002) demonstrated that biostimulation with methanol and acetate led only to dechlorination of tetrachloroethene (PCE) to cis-1,2-dichloroetene (cis-1,2-DCE). By contrast, the application of the KB-1 consortium enabled the complete conversion of chlorinated ethenes to ethene. Within 200 days, the concentrations of PCE, trichloroethene, and cis-1,2-DCE declined to below 5 μg/L, and mass-balance analysis identified ethene formation as the primary mechanism of depletion. Krett et al. (2024) confirmed these observations and reported corresponding changes in functional markers and microbiome composition. Thus, effectiveness should be judged by completion of the entire transformation pathway rather than by acceleration of early steps alone. In systems where process conditions limit performance, oxygen availability and hydraulic parameters are critical. Da Silva et al. (2020) reported that 1,4-dioxane concentrations did not meaningfully decline under unsuitable conditions, whereas bioaugmentation improved removal when appropriate conditions were maintained. However, this improvement was sensitive to oxygen limitation and adverse hydrodynamics. Evaluation criteria must therefore separate the effect of bioaugmentation from the effect of operational conditions and remain consistent with the intended engineering objective (Tyagi et al., 2011; Herrero and Stuckey, 2015).

For trace contaminants, effectiveness is evaluated by achieving concentrations below analytical detection limits and by demonstrating a measurable reduction in exposure. Li et al. (2023) reported that bioaugmentation with propanotrophic microorganisms reduced 1,4-dioxane to nondetectable levels. For hydrophobic and semi-volatile compounds, including polychlorinated biphenyls (PCBs), assessment must also consider emission control. Bako et al. (2022) reported considerable decreases in PCB mass in the gas phase and emissions of the dominant congener, resulting in lower inhalation exposure. A further distinction is required between analytical effectiveness and toxicological effectiveness. Adrion et al. (2016) observed that surfactants enhanced desorption and increased polycyclic aromatic hydrocarbon (PAH) removal, reaching approximately 80% of quantifiable PAHs in some cases. However, toxicity and genotoxicity increased when compared with controls in several systems. Villaverde et al. (2019) likewise showed that although modifying bioavailability affected pyrene mineralisation, combining such amendments with inoculation did not consistently produce additive effects. Evaluation criteria must therefore specify whether they investigate the effect of bioaugmentation itself, the effect of modified bioavailability, or the combined effect of both and should be supported by structured optimisation of process parameters (Zhao et al., 2020).

### 2.2 Functional persistence: stability under environmental variability

Functional persistence is a key criterion in bioaugmentation assessment and refers to the system’s ability to maintain activity under changing geochemical conditions in the presence of toxic compounds and under competitive pressure. Becker (2006) demonstrated that strains of the genus *Dehalococcoides* do not always dominate during natural attenuation, whereas engineered systems promote their proliferation. The presence of species that catalyse successive dechlorination steps can further enhance chloroethene transformation. Morson et al. (2022) investigated cultures of *Dehalococcoides mccartyi* and confirmed the long-term stability of multi-strain systems. They found no evidence of facile transfer of the genomic island containing the *vcrA* operon, which indicates that stable performance depends on population redundancy and complementarity among populations rather than on a single genetic factor. From a spatial perspective, da Silva et al. (2020) reported sustained detection of the functional marker initiating dioxane degradation (*thmA*) at the inoculation site, with no measurable migration after 90 days. This confinement of activity to the treated area indicates the formation of a localised biobarrier. Thomassin-Lacroix et al. (2002) further demonstrated variability in system response. In their study, total petroleum hydrocarbon (TPH) concentrations declined irrespective of inoculation, and microbial abundance increased similarly in both inoculated and control treatments. These findings indicate that biostimulation, rather than the introduced inoculum, drove the observed changes and demonstrate the need to distinguish between underlying mechanisms (Tyagi et al., 2011; Herrero and Stuckey, 2015). Under field conditions, functional persistence is affected by variations in hydrology, redox conditions, and oxygen availability, particularly in groundwater systems where advection, dispersion, and amendment dilution alter residence time in the reactive zone (Becker, 2006; da Silva et al., 2020; Willmann et al., 2023). Demenev et al. (2022) reported that combining aeration with bioaugmentation reduced TPHs by an average of 73.1%, with reductions reaching up to 95.5% in some locations. These results indicate that functional capacity must be maintained even when pollutants continue to enter the system. Where selective pressure declines as remediation progresses, persistence should be maintained by sustaining the metabolic niche rather than by forcing indefinite inoculum survival. Practical measures include repeated or pulsed inoculation, provision of electron donors or co-substrates that maintain pathway induction, and retention strategies such as immobilisation or localised biobarriers that reduce washout from the reactive zone (Suja et al., 2014; da Silva et al., 2020; Sites, 2021; Demenev et al., 2022; Helmy and Kardena, 2024). Kim et al. (2026) provided an experimentally validated example of the long-discussed concept that stable bioaugmentation depends on an integrated microbial network, in which interdependent populations sustain pathway expression under changing conditions, rather than on the persistence of a single dominant degrader.

### 2.3 Risk reduction: protective outcomes and exposure pathways

A third dimension in evaluating bioaugmentation, particularly relevant for highly toxic contaminants, is risk reduction. Risk reduction refers to a measurable decrease in hazards to ecosystems and human health, not simply a decline in the concentration of the parent compound. Davie-Martin et al. (2017) demonstrated that bioremediation of soils contaminated with PAHs reduced overall cancer risk, but posttreatment risk levels remained above acceptable thresholds. This finding indicates that the degradation of carcinogenic fractions alone does not ensure that protective targets are met. For PCBs, risk reduction may primarily depend on lowering emissions to air, thereby reducing inhalation exposure. Decreases in emissions observed during bioaugmentation should therefore be interpreted as changes in exposure conditions rather than solely as indicators of process performance (Bako et al., 2022). For pharmaceutical micropollutants and antibiotics, assessment must distinguish between chemical transformation and complete mineralisation and must also consider microbiological consequences. Changes in community structure become a risk when they disrupt core ecosystem or treatment functions, enrich resistant or opportunistic populations, alter trophic interactions, or reduce resilience to subsequent disturbance. In sediments and aquatic environments, bioaugmentation targeting such compounds should therefore be evaluated not only by reductions in contaminant concentration but also by its effects on microbial communities and ecological risk (Hong et al., 2020; Chen X. et al., 2025).

A similar issue arises in treatment systems, where toxicity and microbiome responses require concurrent monitoring. In aerobic granular sludge systems, Costa et al. (2025) showed that pharmaceuticals can alter microbial morphology and metabolic activity and can modify effluent toxicity. In this context, biological stability refers to preservation of the microbial organisation and activity required to sustain the intended treatment function, including granule integrity, biofilm cohesion, metabolic cooperation, and resistance to performance collapse. These findings also indicate that bioaugmentation in engineered systems can trigger unanticipated community interactions and impair functions not directly targeted by the intervention, including the stability of biofilm-based treatment and the efficiency of water purification. Another crucial consideration is that bioaugmentation may, in some cases, increase the bioavailability of a toxic compound, enabling its uptake or extraction. Abou-Shanab et al. (2022) demonstrated that inoculation with arsenic-transforming bacteria increased metal uptake by 12–43% compared with controls, with maximum accumulation reaching 1.9 g/kg dry leaf mass in the hyperaccumulator *Pteris vittata*. In such cases, risk reduction does not necessarily depend on an immediate decrease in soil concentration but on more efficient removal of contaminants from the system, provided that harvested biomass is properly managed. The risk dimension also includes the stability of treatment functions under stress. Process failure can generate secondary hazards, including the formation and release of intermediates, increased effluent toxicity, or sustained exposure. Yang et al. (2025) reported that some micropollutants can impair key biological pathways and disrupt treatment performance, whereas bioaugmentation with resistant strains maintains removal efficiency.

### 2.4 Implications of multi-criterion evaluation

No single criterion is sufficient to define bioaugmentation success. Removal efficiency may be high while persistence is poor, and concentration decline may occur without a corresponding reduction in toxicity or exposure. In practice, the three criteria should be prioritised according to the primary decision context. Where toxic intermediates are plausible, pathway completion and product chemistry should be weighted first. Where contaminant loading is prolonged or hydrogeochemical variability is expected, persistence should be weighted first. Where the principal management aim is reduction of exposure or ecological effect, toxicity, emission, and bioeffect endpoints should take precedence over concentration decline alone. The purpose of the framework is therefore not to collapse these criteria into a single score, but to force an explicit statement of which endpoint governs interpretation at a given site (Becker, 2006; Adrion et al., 2016; Davie-Martin et al., 2017; Bako et al., 2022; Krett et al., 2024). Table 1 summarises these three evaluation axes by specifying their scope, the main lines of evidence, the common sources of misinterpretation, and the essential reporting elements.

**Table 1:** Multi-criterion evaluation for field bioaugmentation.

Evaluation axis	Scope	Core lines of evidence to prioritise	Typical limitations affecting the interpretation of results	Essential elements of reporting and quality assurance	Reference
Functional performance	Degree to which the intended function is delivered, for example pathway progression, transformation completeness, process stabilisation and exposure flux change where relevant	Substrate product logic confirmation of pathway closure to lower hazard end products and alignment with the engineering objective such as removal or emission reduction	Treating concentration decline as evidence of detoxification, overlooking intermediate accumulation and incomplete pathways, and conflating enhanced contact or bioavailability with biological transformation	The performance endpoint should be defined for pathway closure, emission reduction and stabilisation outcome, and operational conditions that affect expression such as redox, oxygen, donors, acceptors and co substrates should be documented	Adrion et al. (2016), Bako et al. (2022), Bradley and Chapelle (2010), Herrero and Stuckey (2015), Smidt and de Vos (2004)
Functional persistence	Maintenance of functional activity and effect under environmental variability such as hydrology, redox dynamics, oxygen, competition and selective pressure	Time resolved functional markers of capacity and activity, persistence within the reactive zone, resilience under fluctuating boundary conditions and evidence for stable interdependencies	Assuming initial success implies durability, ignoring spatial limitation such as localised biobarrier behaviour, and interpreting gene presence without energetics as persistence	Monitoring horizons should be predefined, capacity should be distinguished from activity, and spatial distribution of function should be mapped against transport processes	Aldas-Vargas et al. (2024), Becker (2006), Ciric et al. (2010), Mansouri et al. (2022), Sites (2021)
Risk reduction	Change in hazard exposure profile, including toxicity outcomes, exposure routes, unintended mobilisation and microbiome consequences	Integration of transformation products with bioassays and biological effect assessment, exposure focused metrics such as emission flux where relevant and microbiome or AMR indicators when chemicals exert selective pressure	Analytical effectiveness not translating to reduced toxicity, increases in exposure after mobilisation, and neglect of AMR related outcomes in antibiotic and WWTP contexts	Risk endpoints should be specified for toxicity, exposure route and AMR indicators, and the linkage between risk logic and monitored outputs such as products, bioassays and microbiome should be demonstrated	Chen et al. (2024), Davie-Martin et al. (2017), Fenner et al. (2013), Raper et al. (2018), Uluseker et al. (2021)

AMR, antimicrobial resistance; WWTP, wastewater treatment plant. The table presents field bioaugmentation as a form of microbiome engineering evaluated across three independent success criteria, namely functional performance, functional persistence and measurable risk reduction. It highlights that concentration based reporting alone cannot provide robust inference, and that credible evaluation requires integrating pathway chemistry, functional and activity evidence and process context while explicitly addressing the trade-offs across these axes.

## 3 Microbiological determinants of bioaugmentation performance

A proper operational understanding of bioaugmentation requires attention to two interconnected levels. The first is the biochemical level, which concerns the presence and activity of specific degradation pathways. The second is the ecological level, which governs whether the introduced function is maintained within a dynamic microbial community. From this perspective, bioaugmentation constitutes a functional intervention intended to introduce or reinforce a defined metabolic capability in a stable manner, thereby affecting both the rate and completeness of detoxification (Lyon and Vogel, 2011; Tyagi et al., 2011; Herrero and Stuckey, 2015; Chettri et al., 2024). Its effectiveness depends on meeting the environmental and operational conditions necessary for enzymatic activity and for incorporation of the introduced function into the resident microbiome (Gao et al., 2022; Mansouri et al., 2022; Muter, 2023). Clarifying the mechanism therefore requires joint analysis of pathway progression, microbial community response, and relevant process conditions (Nandy et al., 2021; Sites, 2021; Mansouri et al., 2022; Li et al., 2026).

A central determinant of mechanism is whether the degradation pathway proceeds in the correct direction and reaches completion, thereby producing a nontoxic end product. Studies confirm that full transformation requires sustained populations capable of catalysing the terminal reaction steps, together with environmental conditions that permit their continued activity (Smidt and de Vos, 2004; Becker, 2006; Heavner et al., 2018; Ortiz-Medina et al., 2023; Krett et al., 2024). Verification must therefore extend beyond detection of relevant microorganisms to confirmation of enzyme expression and *in situ* activity because persistence of function depends on demonstrated activity rather than mere genetic presence (Mansouri et al., 2022; Varsha et al., 2022; Sharma et al., 2024; Salam and Obayori, 2026). Completion of the pathway also depends on environmental structure, including redox conditions, electron donor availability, oxygen supply, and cosubstrate presence. Studies across systems treating aromatic compounds and addressing contaminant mobilisation indicate that pathway closure depends on appropriate redox stratification and fulfilment of induction conditions for degradative enzymes (Deng et al., 2022; Gao et al., 2022; Bertolini et al., 2023; Li et al., 2023; Muter, 2023). Transport and bioavailability processes are equally decisive because they control physical contact between microorganisms and contaminants and can determine environmental outcome independently of intrinsic enzymatic capacity (Adrion et al., 2016; Villaverde et al., 2019; Ferraro et al., 2021; Ferraro et al., 2022; Wang et al., 2026). Table 2 summarises the mechanistic domains that determine whether introduced function is expressed, maintained, or lost under field conditions.

**Table 2:** Mechanistic factors shaping bioaugmentation success and decline.

Mechanistic domain	Primary determinants	Observable signatures (general)	Common failure modes	Design levers to reduce failure risk	Reference
Pathway biochemistry	Directionality and completeness of transformation; presence of terminal step capability; induction and expression constraints	Product formation consistent with pathway closure; congruence between functional markers and expected intermediates and end products	Stalling at intermediates; apparent removal without pathway closure; mismatch between inoculum function and limiting step	Selection of functional modules targeting rate-limiting or terminal steps; ensuring that induction conditions and energetics are feasible	Bradley and Chapelle (2010), Heavner et al. (2018), Krett et al. (2024), Smidt and de Vos (2004),
Energetics and redox architecture	Electron donor and acceptor availability; redox zoning; oxygen supply constraints	Coupled shifts in redox indicators and functional activity; sustained activity only within suitable zones	Loss of function after boundary condition shifts; competition for reducing equivalents; oxygen limitation undermining aerobic functions	Engineering of redox zones; management of donor and acceptor delivery; alignment of reactor or hydrogeological architecture with the target metabolism	Bertolini et al. (2023), Herrero and Stuckey (2015), Ortiz-Medina et al. (2023)
Bioavailability and mass transfer	Sorption and desorption constraints; contact between biomass and contaminant; mobilisation effects	Disparity between nominal potential and realised rates; sensitivity to mixing, porosity and dispersion	Loss of function after boundary condition shifts; competition for reducing equivalents; oxygen limitation undermining aerobic functions	Coupling of bioavailability engineering with risk checks; optimisation of contact strategy including carrier, mixing and delivery	Adrion et al. (2016), Semple et al. (2003), Villaverde et al. (2019)
Microbiome ecology	Colonisation, competition, selection, succession; redundancy and complementarity	Coherent community restructuring consistent with the functional niche; persistence tied to selective pressure	Rapid dilution and competitive exclusion; decline of function under weak selective pressure; reliance on a single population	Use of consortia and functional redundancy; maintenance of selective pressure; design of retention mechanisms	Aldas-Vargas et al. (2024), Kim et al. (2026), Morson et al. (2022), Sites (2021)
Spatial ecology of the reactive zone	Localisation *vs* migration; retention in porous media; dispersal control	Strong activity at the application zone with limited spread; local barrier behaviour	Benefits confined to a small zone; misinterpretation as site-wide improvement	Delivery and retention engineering; immobilisation; recirculation strategies; spatial monitoring	Hong et al. (2020), Sites (2021), Willmann et al. (2023)

The table synthesises the mechanistic logic of the review and shows that field bioaugmentation outcomes reflect the combined constraints of pathway biochemistry, energetics and redox architecture, contaminant bioavailability and transport, and microbiome ecology. It summarises how apparent fade out frequently results from shifts in boundary conditions, insufficient retention or selective pressure, and mass transfer dominance, highlighting the need to engineer the reactive zone rather than treat inoculation as a single event.

Ecological processes, such as colonisation, competition, and long-term population stability, strongly affect bioaugmentation performance because they determine whether introduced functions are maintained over time. Studies indicate that sustained activity more often results from diversity and functional complementarity among populations than from the dominance of a single strain (Morson et al., 2022). When contaminant availability declines, degradative capacity may also decrease, even if initial removal was effective (Aldas-Vargas et al., 2024).

Other studies show that transformation outcomes are linked to changes in ecological niche structure, highlighting the roles of selection and succession (Chen et al., 2021; Jia et al., 2021). The effect of niche conditions is especially clear in extreme environments, where effectiveness depends on selecting organisms that can remain active under demanding conditions (Feng et al., 2020; Kansour and Al-Mailem, 2023; Werheni Ammeri et al., 2023a; Werheni Ammeri et al., 2023b; Sethi et al., 2025).

For pharmaceutical micropollutants, the key issue is not resistance to pharmaceuticals in general, but selection for antimicrobial resistance when the compounds are antibiotics, or when they exert antibiotic-like selective effects. Non-antibiotic pharmaceuticals may still alter microbiome structure and treatment performance, so assessment should distinguish resistance-related risk from broader ecological disturbance (Chen et al., 2024; Chen X. et al., 2025; Nath, 2026).

## 4 Engineering microbial function in environmental systems

Engineering-focused bioaugmentation design translates the concept of inoculation into specific decisions that ensure the introduced function remains available, active, and stable under field or reactor conditions (Lyon and Vogel, 2011; Tyagi et al., 2011; Herrero and Stuckey, 2015; Sites, 2021; Chettri et al., 2024; Fujikawa et al., 2025). A central step involves selecting an inoculum as a defined biochemical module, pathway, or enzyme system, together with its energetic and ecological requirements, in a manner consistent with the characteristics of the target matrix (Lyon and Vogel, 2011; Herrero and Stuckey, 2015; Morson et al., 2022). In practice, this requires identification of the rate-limiting step in the transformation pathway and introduction of a function that allows completion of the pathway to a lower-risk end product rather than simply accelerating early reactions (Heavner et al., 2018; Krett et al., 2024). Selection of inoculum should begin with the transformation mechanism. Growth-linked mineralisation, cometabolic conversion, and syntrophic pathway closure impose different design constraints. For chloroethenes, the decisive question is whether the introduced population can sustain terminal dechlorination to ethene under the available donor supply and redox regime, rather than only accelerate the first reduction step (Becker, 2006; Grostern and Edwards, 2006; Bradley and Chapelle, 2010; Heavner et al., 2018; Peng et al., 2019). For antibiotics and many pharmaceutical micropollutants, removal is often cometabolic or partial, so design must verify mineralisation and assess microbiome and resistance-related consequences rather than relying on parent-compound loss alone (Uluseker et al., 2021; Li et al., 2022; Clarke et al., 2024; Lopez Gordillo et al., 2024). For pesticides and hydrophobic contaminants, the limiting step may lie in enzyme induction, co-substrate supply, or contaminant accessibility, which makes bioavailability management part of inoculum design rather than an ancillary addition (Sharma et al., 2014; Cycoń et al., 2017; Kumar et al., 2018; Villaverde et al., 2019; Lopes et al., 2022; Sandhu et al., 2022; Yu et al., 2025). Where competition for electron donors constrains pathway closure, the selected strategy must also match the energetic requirements of the target metabolism (Yu et al., 2006). This principle is clearly illustrated in the remediation of chlorinated ethenes, where the objective is full reductive dechlorination. Achieving this outcome depends on maintaining populations capable of catalysing the terminal steps under appropriate redox and oxygenation conditions (Heavner et al., 2018; Krett et al., 2024; Ortiz-Medina et al., 2023). Field studies confirm that effective bioaugmentation depends on selecting an inoculum capable of completing the transformation pathway, rather than focusing only on reduction of the parent compound (Heavner et al., 2018). Effective design should integrate inoculation with targeted biostimulation, meaning adjustment of the environmental conditions required for the selected pathway, such as electron donor or acceptor supply, oxygen regime, redox control, or provision of co-substrates, and with monitoring of pathway-specific functional markers. Functional persistence is required over the operational horizon needed to reach remediation endpoints, not as indefinite survival of the inoculum after those endpoints have been achieved. Because field projects are constrained by time, cost, and sampling access, the evidence package should be tiered: a minimum dataset should include product chemistry, process conditions, and a small panel of pathway-specific markers, whereas broader omics and predictive modelling should be reserved for cases in which targeted monitoring cannot resolve attribution or safety (Zhao et al., 2020; Sites, 2021; Mansouri et al., 2022; Krett et al., 2024).

### 4.1 Inoculum selection: functional modules over single-strain approaches

Effective bioaugmentation design begins with defining the intended function: initiation of a pathway, completion of a pathway, or provision of supporting functions such as cosubstrate supply, stabilisation of redox conditions, or mobilisation of hydrophobic contaminants. Many applications therefore employ natural, enriched, or synthetic consortia capable of addressing kinetic, environmental, and ecological constraints in parallel. Functional redundancy within such consortia contributes to sustained activity over time. Consortia are generally preferable when pathway closure depends on metabolic division of labour, fluctuating redox microzones, or protection against competitive loss of a single strain (Lyon and Vogel, 2011; Nandy et al., 2021; Chettri et al., 2024).

Research increasingly indicates that performance depends not only on the choice of inoculum but also on the selection of suitable energetic conditions. Expression of degradative functions and integration of introduced microorganisms into the ecosystem require an appropriate balance of electron donors and acceptors (Kim et al., 2026). In many environmental matrices, limited bioavailability or unfavourable conditions for sustained pathway expression, rather than absence of catalytic potential, constrain effectiveness. Thus, inocula are often designed as functional modules in which the primary degrading organism is combined with organisms or components that enhance contaminant accessibility or stabilise system conditions (Alvarez et al., 2022). In petroleum hydrocarbon remediation, degradative strains are frequently paired with agents that increase the availability of hydrophobic fractions, including biosurfactants used as active functional components rather than simple additives (Abis et al., 2025; Baghaie and Keshavarzi, 2025). Inoculum design therefore encompasses both microbial characteristics and the environmental factors governing contaminant accessibility, which together determine whether the contaminant can be effectively utilised (Klim et al., 2025).

Physiological compatibility with the target matrix is also critical. In saline or other extreme environments, effectiveness depends on the capacity of introduced organisms to remain metabolically active under harsh conditions, which directly affects stability of the degradative function (Kansour and Al-Mailem, 2023). In pesticide remediation, consortia are selected not only for their degradation capacity but also for their ability to withstand ecological pressures, including competition, seasonal variation, and selective stress (Anwar et al., 2024; Faridy et al., 2024; Sethi et al., 2025). Design must further consider spatial heterogeneity of contamination. In zones with high contaminant concentrations, inoculum performance may be limited, and alternative or supplementary measures may be required (Papadopoulou et al., 2018).

### 4.2 Delivery strategies: distribution, retention, and effective biological dose

A second key element of bioaugmentation design concerns the method used to apply and deliver the inoculum to the zone where activity is required. In aqueous systems, this includes distributing the inoculum within the target zone, ensuring its retention in the porous medium, and supplying biomass under conditions compatible with metabolism, such as suitable redox potential and availability of electron donors. Field evidence indicates that application strategy directly affects contact between microorganisms and contaminants and can determine reaction rates. It therefore constitutes a design variable, not merely a technical detail (Steffan et al., 1999). In matrices with fixed structure, delivery methods also affect the microenvironment in which microorganisms interact with contaminants and limit uncontrolled dispersion in heterogeneous media. Data further show that carrier selection and inoculum form (e.g., immobilised biomass versus free cells) affect cell retention, local contaminant accessibility, and resilience to environmental fluctuations. These decisions are therefore as critical as strain selection in determining performance (Hong et al., 2020).

In soils and sediments, immobilised inocula, carrier-bound biomass, compost-assisted delivery, and local mixing can improve retention and contact with sorbed contaminants, but they may create steep microscale gradients, increase amendment demand, and hinder uniform distribution. In groundwater, direct injection and recirculation are suited to plume-scale treatment, but dilution, short residence time, and preferential flow can disperse cells beyond the reactive zone. Permeable biobarriers and carrier-assisted retention improve localisation, yet they also restrict the treated volume and may require repeated amendment or reinoculation (Steffan et al., 1999; Hong et al., 2020; Sites, 2021; Willmann et al., 2023).

From a design perspective, biological dose, defined as the number of viable cells introduced into the matrix, is a critical parameter. Evidence indicates that optimal dosing depends on contamination age and the availability of readily degradable fractions; inoculation strategies must therefore be adapted to the contamination history (Silva et al., 2015; Viegas et al., 2019). At field scale, application strategy cannot be separated from the supply of oxygen or other electron acceptors because distribution and oxygenation often represent the main limiting factors. Field experience indicates that performance depends on coordinated design of inoculum delivery, oxidant supply, and management of the reactive zone. These factors determine whether bioaugmentation functions as a stable biobarrier or produces only a localised effect (Demenev et al., 2022) (Figure 1).

### 4.3 Sustaining function: selective pressure, retention mechanisms, and monitoring

A third core element of bioaugmentation design concerns maintaining function over the long term. This requires measures that prevent the observed effect from remaining transient. Such measures include maintaining selective pressure through appropriate energetic and redox conditions; ensuring biomass retention by means of carriers, reactive barriers or recirculation; planning redosing when necessary; and implementing biomonitoring capable of detecting functional decline before it appears in contaminant concentrations (Tyagi et al., 2011; Herrero and Stuckey, 2015; Mansouri et al., 2022). In systems where selective pressure is weak, introducing a suitable inoculum does not ensure persistence (Aldas-Vargas et al., 2024; Sethi et al., 2025). Biomonitoring is therefore increasingly incorporated as a design component. It integrates chemical analyses with toxicity indicators and molecular or functional markers selected based on system characteristics and project objectives (Ciric et al., 2010; Mansouri et al., 2022; Varsha et al., 2022). For TPH systems, useful monitoring combinations include hydrocarbon fraction profiles together with respiration or oxygen-demand measurements and hydrocarbon-catabolic markers. For PAHs, transformation-product profiling should be paired with toxicity and genotoxicity bioassays. For chlorinated ethenes, substrate-product balances and reductive dehalogenase markers provide the clearest evidence of pathway closure (Bradley and Chapelle, 2010; Ciric et al., 2010; Adrion et al., 2016; Mansouri et al., 2022; Krett et al., 2024). At field scale, microbial community analysis helps distinguish true biodegradation from dilution or transport when shifts in community structure co-occur with increases in pathway-specific markers or transcripts, growth of the intended functional guild within the reactive zone, and product formation consistent with the expected pathway. In practice, a unique inoculum signature is easiest to detect when the inoculum carries distinctive functional markers, whereas taxonomic tracking alone is often unreliable because introduced taxa may disperse, overlap with resident populations, or fall below detection (Ciric et al., 2010; Sites, 2021). In soil and composting systems, long-term activity depends on stabilising physical parameters and establishing microenvironments that retain biomass. Combining localised bioaugmentation with biostimulation can sustain high activity and abundance of degrading microorganisms, indicating that durable performance depends on continued system support rather than on a single inoculation event (Suja et al., 2014; Helmy and Kardena, 2024).

### 4.4 Optimisation and transferability: from treatability tests to data-driven design

A bioaugmentation strategy should remain internally consistent with the range of environmental conditions expected at the target site. Inoculum selection should correspond to the energetic and redox requirements of that site, the method of application should fit its bioavailability and mass-transfer constraints, and the maintenance plan should reflect expected selective pressures, dispersal, and microbial competition. In practice, this alignment is established through staged testing to assess feasibility, identify key limitations, and confirm performance at pilot or field scale (Kuppusamy et al., 2016; Nandy et al., 2021; Sites, 2021). Design is increasingly informed by structured optimisation methods, including systematic selection of process parameters, which is particularly relevant when separating inoculum-driven effects from those attributable to system configuration (Zhao et al., 2020). Machine-learning and bioinformatics tools are also used to identify performance indicators and support prediction, provided that modelling is based on biological and operational data rather than on statistical associations alone (Ashkanani et al., 2024; Siddique et al., 2025; Zhang et al., 2025). Before deploying bioaugmentation, site assessment should first determine whether natural attenuation or biostimulation alone can achieve pathway closure within the required time frame. Bioaugmentation is justified when catalytic capacity is missing, when the terminal step is absent or unstable, or when field conditions repeatedly suppress the required function despite targeted stimulation. This tiered logic is important because remediation projects rarely permit unlimited sampling, analysis, or reinoculation.

All of these approaches share a common principle: bioaugmentation design must be treated as an evidence-based process. Decisions regarding inoculum selection, formulation, and maintenance strategy should be guided by a defined operational hypothesis and supported by a monitoring plan that allows reliable evaluation of outcomes. Field studies on chloroethenes demonstrate that, without staged implementation and monitoring of functional markers, it is difficult to differentiate true detoxification from temporary changes in contaminant distribution (Heavner et al., 2018; Ortiz-Medina et al., 2023; Krett et al., 2024). Similarly, examples from soil and sludge systems indicate that carriers, immobilisation techniques, and measures enhancing bioavailability can become integral elements of inoculum design when their function is clearly connected to the underlying mechanism and incorporated into the monitoring framework (Hong et al., 2020; Alvarez et al., 2022; Bako et al., 2022; Abis et al., 2025).

## 5 Matrix-dependent constraints on inoculum establishment

The effectiveness of bioaugmentation largely depends on the conditions that govern contact between the contaminant and biomass and on local redox conditions. The properties of the environmental matrix affect the rate, completeness, and persistence of transformation. An inoculum that performs well under laboratory conditions may thus be limited in the field by sorption, diffusion constraints, and system heterogeneity, making the translation of laboratory findings to field scale both biological and engineering challenges (Nandy et al., 2021; Sites, 2021; Aldas-Vargas et al., 2024; Das et al., 2025). From an engineering perspective, bioaugmentation must be evaluated in terms of the specific matrix and technological configuration rather than as a simple relationship between a contaminant and degrading organism. Table 3 consolidates the matrix-specific constraints, meaningful performance endpoints, persistence vulnerabilities, and monitoring priorities that determine transferability across environmental compartments. Factors such as contact efficiency, ecological niche stability, and mass-transfer limitations can determine performance more strongly than the catalytic potential of microorganisms themselves (Lyon and Vogel, 2011; Sayara et al., 2011; Bako et al., 2022; Krett et al., 2024). This perspective indicates that bioaugmentation strategies must be adapted to the specific characteristics of the target environment and the practical conditions of implementation. Such adaptation is necessary to achieve both effective performance and long-term stability of the technology.

**Table 3:** Matrix- and configuration-informed design considerations for translating laboratory performance to field conditions.

Matrix/Configuration context	Dominant constraints (general)	Appropriate performance endpoints	Persistence vulnerabilities	Monitoring emphasis	Reference
Soils with hydrophobic contaminants	Sorption and limited bioavailable fraction; diffusion constraints; heterogeneity	Pathway completion and reduction in relevant exposure proxies; coherence between removal and biological effect	Seasonal variability; weak selective pressure; amendment driven mobilisation altering exposure	Bioavailability indicators; transformation product profiling; paired bioassays; functional markers	Adrion et al. (2016), Davie-Martin et al. (2017), Semple et al. (2003)
Sediments acting as emission sources	Solid phase sequestration coupled to release processes; flux driven exposure	Exposure flux reduction where it governs risk; confirmation of transformation mechanism	Spatial confinement; re-release after disturbance; incomplete transformation under suboptimal conditions	Flux and release oriented metrics with mechanistic markers; product tracking	Bako et al. (2022), Raper et al. (2018)
Groundwater/aquifers	Geochemical heterogeneity; transport and dilution; maintaining the redox regime in porous media	Verified pathway closure to lower risk end products; stability of reactive zone function	Dilution of amendments; shifting redox; localisation without plume wide benefit	Substrate-product balance; functional and activity markers; process condition logging	Becker (2006), Major et al. (2002), Smidt and de Vos, 2004
Wastewater/sludge treatment systems	Complex mixtures; operational variability; microbiome as process infrastructure	Combined outcome covering removal and resilience or stability of core process functions; risk reduction extending beyond the decline of the parent compound	Process disturbances under stressors; selective pressure and AMR dynamics	Integrated chemistry, bioassays and microbiome or AMR indicators; stability metrics	Costa et al. (2025), Pickering et al. (2024), Uluseker et al. (2021), Yang et al. (2025)
Hybrid systems (bioaugmentation plus sorption/phytoremediation)	Mechanism blending; attribution complexity; compartment transfer risks	Exposure reduction with verified transformation and controlled fate	Shifts in partitioning masquerading as biodegradation; delayed risks via secondary compartments	Attribution focused monitoring; fate tracking across compartments; biological effect endpoints	Fenner et al. (2013), Werheni Ammeri et al. (2023a), Werheni Ammeri et al. (2023b),

AMR, antimicrobial resistance. This table consolidates a matrix-oriented view of bioaugmentation and highlights that dominant constraints and meaningful endpoints vary across environmental compartments and technological configurations. It aligns evaluation with exposure pathways and system architecture, supporting transferability by clarifying which constraints are likely to prevail outside controlled laboratory conditions.

Soils contaminated with hydrophobic compounds are a classical matrix in which mass-transfer limitations and sorption processes jointly affect bioaugmentation performance. In such systems, the rate at which contaminants become bioavailable often governs the overall biodegradation potential of microorganisms (Semple et al., 2003). Studies conducted under composting conditions indicate that careful control of aeration and provision of readily available organic matter may affect process efficiency more than inoculation itself (Lyon and Vogel, 2011; Sayara et al., 2011). This observation has direct implications for selecting ex situ configurations and defining operational parameters. Ex situ and *in situ* configurations differ in their control over contact and mass transfer. Ex situ systems provide intensified mixing and transfer, whereas in situ applications are limited by diffusion barriers and micro-heterogeneity in the matrix. These differences have encouraged the use of mixed configurations and the development of anaerobic approaches adapted to the prevailing redox conditions (Ferraro et al., 2021; Nandy et al., 2021; Ferraro et al., 2022; Wang et al., 2026). Strategies that increase bioavailability can substantially accelerate contaminant removal. However, they may also alter toxicity patterns and exposure pathways, which requires balanced evaluation of both benefits and potential risks associated with mobilisation (Adrion et al., 2016; Villaverde et al., 2019; Semple et al., 2003). In agricultural soils, seasonal variation, aggregate structure, and shifting competitive pressures within the microbiome limit direct extrapolation of results obtained in simplified mineral substrates. Therefore, a parallel assessment in controlled media and actual soil systems is advisable to optimise configuration and define an appropriate monitoring period (Sites, 2021; Książek-Trela et al., 2025).

Evaluation of soil and sediment remediation increasingly combines chemical concentration data with risk-based indicators. Davie-Martin et al. (2017) showed that although bioremediation of PAH-contaminated soils reduced health risk, residual risk levels did not always meet accepted thresholds. This finding confirms that final assessment should be based on demonstrable risk reduction rather than concentration decline alone (Juhasz and Naidu, 2000). Studies of compost-based systems further indicate that, under field conditions, operational parameters such as aeration, moisture, organic matter content, and porosity often determine overall performance more strongly than inoculation. This effect is particularly evident in weathered soils with limited contaminant bioavailability. The application of structure-forming materials, including biocompost, illustrates that technological configuration may become the primary determinant of remediation efficiency in matrices where mass transfer limits transformation (Helmy and Kardena, 2024).

In sediments and sludge, which both accumulate contaminants and serve as emission sources, assessment of bioaugmentation should prioritise reduction of release flux rather than relying solely on changes in solid-phase concentrations because flux determines exposure (Bako et al., 2022). Studies on PCBs indicate that decreases in gas-phase emissions may provide a more meaningful indicator of effectiveness than concentration decline alone, and that concurrent monitoring of functional markers is necessary to verify the underlying mechanism (Bako et al., 2022). Findings for highly toxic congeners further indicate that performance depends not only on the presence of suitable degradative microorganisms but also on environmental conditions that permit the expression of enzymes initiating the reaction (Sandhu et al., 2022). Process design must therefore integrate biological components with environmental physicochemistry because outcomes in sediments and soils are governed by the interaction among sorption, desorption and biotransformation rather than by any single parameter (Semple et al., 2003; Bako et al., 2022). Comparable considerations apply to antibiotic-contaminated sediments, where evaluation of chemical removal should be accompanied by ecological risk assessment, including analysis of selective pressure and effects on microbiome structure and function. For this reason, strategies based on synthetic consortia and risk-oriented evaluation are increasingly used in place of simple comparisons between inoculated and control systems (Chen X. et al., 2025).

In aquifers and groundwater systems, bioaugmentation performance is constrained by geochemical heterogeneity and solute transport within the porous matrix. Even a highly specialised inoculum will not remain active unless it reaches the reactive zone and the redox conditions required for completion of the degradation pathway are maintained (Becker, 2006; Krett et al., 2024). Field investigations show that full transformation of contaminants depends on a stable reducing environment and confirmation that the end product presents lower ecological risk (Smidt and de Vos, 2004; Major et al., 2002). Accordingly, monitoring of functional markers and evaluation of biodegradation potential after cessation of active amendment are increasingly emphasised. Aquifer systems are dynamic and subject to dilution through transport processes and fluctuations in geochemical conditions, which can diminish or obscure the observed effect (Krett et al., 2024).

For groundwater systems treating chlorinated hydrocarbons, pathway verification remains essential because concentration decline can mask persistence of intermediates (Smidt and de Vos, 2004; Scheutz et al., 2008; Wang et al., 2015). In wastewater matrices containing pharmaceutical micropollutants, controlled studies show that selected inocula can accelerate removal, but translation to real wastewater remains inconsistent because matrix complexity suppresses mineralisation and weakens reproducibility (Marco-Urrea et al., 2010; Falås et al., 2013; Dalecka et al., 2021; Maqsood et al., 2024). These observations reinforce the central point of this section: matrix constraints, not inoculum identity alone, govern field performance.

## 6 Monitoring, attribution, and validation of inoculum-driven effects

Monitoring of bioaugmentation is meaningful only if it addresses two problems: whether transformation results in genuine detoxification and whether the observed effect is attributable to the introduced inoculum rather than to environmental changes, dispersion, or stimulation of the indigenous microbiome (Tyagi et al., 2011; Fenner et al., 2013; Herrero and Stuckey, 2015). Reliance on a single metric, such as reduction in concentration, does not allow clear attribution and may conceal increased risk associated with intermediate formation or mobilisation of bioavailable fractions (Davie-Martin et al., 2017; Raper et al., 2018). Monitoring design (Figure 1) should therefore follow a system-level approach. It should include process parameters, assessment of contaminant fate, analysis of functional markers and, where appropriate, direct measures of biological effect (Ciric et al., 2010; Demenev et al., 2022; Mansouri et al., 2022). Table 4 summarises the minimum monitoring package required to support both causal attribution and control of secondary risk in field bioaugmentation. The most reliable basis for attributing effect remains the substrate-product balance, where evidence of pathway closure and formation of a lower-risk end product is decisive (Smidt and de Vos, 2004; Bradley and Chapelle, 2010). Field investigations of dechlorination demonstrate that only simultaneous tracking of contaminant decline, accumulation of the terminal product, and stability of environmental conditions allows true biodegradation to be distinguished from apparent removal (Raper et al., 2018; Major et al., 2002). Chemical mass balance is increasingly supplemented by mechanistic evidence derived from functional markers and activity-based measurements. These data demonstrate whether growth and activity of populations responsible for terminal reaction steps account for the observed concentration decrease, rather than changes arising from redistribution of the compound (Smidt and de Vos, 2004; Bradley and Chapelle, 2010; Krett et al., 2024). It has also been demonstrated that detection of a relevant gene is insufficient evidence in the absence of suitable energetic conditions. Monitoring of process parameters is therefore essential within the overall chain of evidence (Ortiz-Medina et al., 2023).

**Table 4:** Risk-centric monitoring package for safety, secondary risks and credible effect attribution.

Risk/Attribution focus	Required demonstration (general)	Minimum monitoring elements	Secondary risks to control	Interpretation safeguards	Reference
Causal attribution to inoculation	Observed outcome reflects the introduced function rather than process condition change, dilution, dispersion or stimulation of autochthonous microbiota	Substrate-product balance; functional markers linked to the intended step; process condition tracking	Misattribution under co interventions including donors, aeration and surfactants; false positives arising from gene presence	Use of multi-line evidence with cross validation; separation of bioaugmentation and biostimulation contributions	Bradley and Chapelle (2010), Ciric et al. (2010), Mansouri et al. (2022)
Transformation products and detoxification	Transformation proceeds towards lower hazard end products; intermediates do not increase hazard	Identification of transformation and mineralisation products; evidence of pathway completeness; biological effect assessment	Hazard profile shift due to intermediates; toxicity increase despite parent decline	Reporting of both chemical and biological endpoints with explicit interpretation of removal versus detoxification	Adrion et al. (2016), Davie-Martin et al. (2017), Fenner et al. (2013)
Bioavailability engineering and exposure	Mobilisation does not raise exposure risk beyond acceptable bounds	Bioavailability proxies; exposure relevant metrics including flux when relevant; coupled bioassays	Elevated exposure *via* mobilisation; non additive interactions with inoculum	Treatment of mobilisation as a risk relevant intervention with use of exposure focused endpoints	Bako et al. (2022), Raper et al. (2018), Semple et al. (2003)
AMR and microbiome consequences	Intervention does not exacerbate resistance selection or transmission and does not destabilise functional microbiomes	ARG and MGE profiling where justified; selective pressure indicators; microbiome structure and function response	Resistance selection hotspots including treatment systems; community disruption undermining process integrity	Interpretation of AMR outputs in parallel with chemical transformation and process stability with distinction between capacity and activity	Chen et al. (2024), Clarke et al. (2024), Pickering et al. (2024), Uluseker et al. (2021)
Data-driven support (AI/ML, MCDA)	Predictions are grounded in mechanistic and monitoring standards, not correlations alone	Standardised monitoring datasets; validated feature sets; explicit uncertainty	Over simplification; spurious correlations; non-transferable models	Use of ML as decision support tied to the evidence package with application of MCDA to prevent single metric optimisation	Ashkanani et al. (2024), Siddique et al. (2025), Zhao et al. (2020)

AI, artificial intelligence; AMR, antimicrobial resistance; ARG, antibiotic resistance gene; MCDA, multi-criteria decision analysis; ML, machine learning. This table specifies a risk-centric monitoring package that supports both safety evaluation and defensible attribution of effects in field bioaugmentation. It integrates product chemistry, functional and activity markers, process condition logging, biological effect assessment and, where relevant, AMR or microbiome indicators, reflecting the review’s emphasis on multi line evidence rather than single-indicator conclusions.

Monitoring and attribution in bioaugmentation should be structured as an integrated body of evidence linking product chemistry, functional and activity markers, and matrix-specific risk indicators, with sufficient temporal and process resolution to distinguish active function from mere genetic or metabolic potential (Major et al., 2002; Ciric et al., 2010; Herrero and Stuckey, 2015; Adrion et al., 2016; Bako et al., 2022; Demenev et al., 2022; Willmann et al., 2023; Lopez Gordillo et al., 2024; Cycoń, 2026).

## 7 Environmental safety and secondary risk

Environmental safety in bioaugmentation cannot be inferred from parent-compound removal alone. A decline in concentration does not, by itself, demonstrate detoxification, because bioaugmentation may alter bioavailability, redirect transformation pathways, change the profile of intermediates and end products, and restructure the receiving microbiome. Safety assessment must therefore integrate chemical, microbiological, and ecological evidence and establish whether the intervention reduces hazard and exposure while preserving key treatment or ecosystem functions (Fenner et al., 2013; Adrion et al., 2016; Davie-Martin et al., 2017; Raper et al., 2018; Bako et al., 2022). This requirement is especially stringent in systems containing antibiotics, where risk arises from both chemical exposure and microbiological consequences. Even when bioaugmentation improves removal, assessment must still address shifts in antibiotic resistance gene profiles, the presence and mobility of mobile genetic elements, and selective pressures operating within the community (Fang et al., 2017; Sabri et al., 2021; Uluseker et al., 2021; Chen et al., 2024; Lopez Gordillo et al., 2024; Chen X. et al., 2025). Mechanistic studies further indicate that bioaugmentation may, in some settings, reduce the potential for resistance transfer by stabilising environmental conditions and limiting oxidative stress, thereby improving process safety (Ren et al., 2021). These effects, however, cannot be assumed and must be demonstrated empirically within the treated system.

A defensible safety assessment also requires distinction between genetic potential and expressed activity. For this reason, monitoring should combine potential-based indicators, including abundance of pathway genes, inventories of reductive dehalogenase genes, antibiotic resistance genes and mobile genetic element copy numbers, metagenome-derived pathway capacity, and predicted metabolic modules, with activity-based measures such as transcriptomics, proteomics, enzyme assays, and transformation-rate measurements (Varsha et al., 2022; Sharma et al., 2024; Salam and Obayori, 2026). This combination is necessary because the mere presence of functional or resistance-related determinants does not establish pathway execution, active resistance selection, or actual environmental risk.

Process integrity forms a second safety dimension. In engineered systems, bioaugmentation may alter microbiome organisation in ways that affect the stability and efficiency of core treatment functions, with direct implications for process control and regulatory acceptability (Pickering et al., 2024; Yang et al., 2025). Predictive and data-driven approaches, including learning-based models, are increasingly used to support this level of assessment, but their value depends on rigorous validation against field observations and operational data (Ashkanani et al., 2024; Siddique et al., 2025; Zhang et al., 2025). Without such validation, prediction cannot substitute for direct evidence of system stability and safe function. Where management or regulatory decisions depend on proof of hazard reduction, chemical data should be accompanied by ecotoxicity endpoints matched to the receiving compartment. Appropriate tools include *Vibrio fischeri* luminescence inhibition, algal growth inhibition, *Daphnia* immobilisation, plant germination and growth assays, earthworm survival, soil enzyme activity, and genotoxicity tests, applied to treated matrices and, where relevant, to inoculum formulations and transformation products. These endpoints are particularly important in PAH, pesticide, sludge, and mixed-contaminant systems, in which concentration decline may diverge from biological response (Silva et al., 2015; Viegas et al., 2019; Brzeszcz et al., 2020; Zaborowska et al., 2020; Zhao et al., 2020).

Safety evaluation should also include the inoculum itself and its ecological footprint after application. This requires genome-based screening for pathogenicity determinants, toxin production potential, transferable resistance loci, and traits capable of disrupting nutrient cycling or treatment performance, together with verification that the inoculum remains confined to the intended treatment zone and does not displace critical resident functions (McDaniel et al., 2007; Sites, 2021; Simmer and Schnoor, 2022; Pickering et al., 2024; Du et al., 2025). Taken together, these considerations indicate that environmental safety in bioaugmentation should be judged not by removal alone, but by demonstrated hazard reduction, preserved process integrity, controlled ecological impact, and clear evidence that secondary risks remain within acceptable bounds.

## 8 Conclusions and future directions

Bioaugmentation should be judged as a controlled intervention aimed at establishing a defined function under site conditions, not as the mere introduction of exogenous microorganisms. Its relevance depends on whether the target activity is expressed where it is needed, maintained for the period required to meet the remediation objective, and linked to a measurable improvement in environmental quality. The presence of the inoculum, taken alone, is not an adequate endpoint.

The central analytical problem is attribution. A decline in parent-compound concentration may reflect true pathway execution, but it may also arise from partial conversion, altered bioavailability, dilution, or stimulation of resident populations. Future studies should therefore define success in operational terms before field application and align monitoring with that decision framework, so that functional expression, persistence, and hazard reduction can be interpreted with sufficient confidence.

Progress in the field will depend on tighter coupling between microbiology and process design. Performance is often limited less by the nominal catabolic capacity of the inoculum than by poor retention, restricted access to the contaminant, unstable redox conditions, or loss of activity under field variability. In practice, inoculum selection, delivery, retention, and maintenance of the metabolic niche should be treated as parts of the same design problem rather than as separate considerations.

Safety must remain integral to evaluation, not secondary to removal efficiency. An intervention cannot be considered successful if it lowers concentration while worsening toxicity, disturbing treatment stability, or introducing unwanted ecological effects. The next phase of bioaugmentation research should therefore focus on clearer causal evidence, better transferability across field settings, and monitoring schemes scaled to the actual decision context. That is the basis on which bioaugmentation can support credible environmental management.
